# Supplementary material for: Lipidomics Reveals Cell Specific Changes During Pluripotent Differentiation to Neural and Mesodermal Lineages
Source: bioRxiv. 2025 Jan 1:2024.12.31.630916. Preprint. [Version 1] doi: 10.1101/2024.12.31.630916 (PMC11722439; doi:10.1101/2024.12.31.630916)
Supplement: Supplementary file 1 [file media-1.pdf]

Supporting Information:

Lipidomics Reveals Cell Specific Changes During Pluripotent Differentiation to Neural and Mesodermal Lineages

Melanie T. Odenkirk,[a] Haley C. Jostes,[b] Kevin Francis, [c,d]* and Erin S. Baker [b]*

[a] Department of Chemistry, North Carolina State University, Raleigh, NC

[b] Department of Chemistry, University of North Carolina at Chapel Hill, Chapel Hill, NC

[c] Cellular Therapies and Stem Cell Biology Group, Sanford Research, Sioux Falls, SD

[d] Department of Pediatrics, University of South Dakota Sanford School of Medicine, Sioux Falls, SD

*** Correspondence:**
Emails: erinmsb@unc.edu and Kevin.Francis@SanfordHealth.org

Table of Contents

Cover page and table of contents **S1-2**

Table S1. Experimental parameters for the Agilent 1290 Infinity II UHPLC............................. **S3**

Table S2. Collision energy ramp for the Agilent 6560 IMS-MS.. **S4**

Tables S3-S18. statistically significant lipid species for each time point comparison........... **S5-31**

Table S1. Lipid liquid chromatography (LC) gradient and column wash for stem cell lipidomic experiments.

	Time (min)	% MPA (60:40 Water: Acetonitrile with 10 mM $NH_4C_2H_3O_2$)	% MPB (90:10 Isopropanol: Acetonitrile with 10 mM $NH_4C_2H_3O_2$)	Flow rate (mL/min)
Lipid elution gradient	0	60	40	0.25
	2	50	50	0.25
	3	40	60	0.25
	12	30	70	0.25
	15	25	75	0.25
	17	22	78	0.25
	19	15	85	0.25
	22	8	92	0.25
	25	1	99	0.25
	34	1	99	0.25
Column wash	34.5	60	40	0.3
	35	1	99	0.3
	35.5	1	99	0.3
	36	60	40	0.35
	37	60	40	0.3
	38	60	40	0.25

Table S2. Ramped IMS-CID fragmentation method

Drift Time (ms)	Collision Energy (eV)
0	0
15	14
19	27
25	45
50	58

Table S3. Statistically significant downregulated lipids for ebs3 versus pluripotent comparison of the neural stem cell lineage.

Lipid	Ionization Mode	p-value	Log_2FC
CL(68:4)	Negative	4.52E-03	-0.520
HexCer(d18:1/24:0)	Negative	3.96E-02	-0.789
HexCer(d18:1/24:1)	Negative	6.03E-03	-0.924
PC(14:0_18:2)	Negative	6.37E-03	-0.576
PE(14:0_16:0)	Negative	7.54E-06	-1.391
PE(14:0_18:1);PE(16:0_16:1)	Negative	1.48E-03	-1.951
PE(16:0_20:4)	Negative	1.32E-04	-1.676
PE(16:1/16:1)	Negative	1.63E-03	-1.568
PE(16:1_18:1)	Negative	1.63E-07	-1.983
PE(18:0_18:3)	Negative	1.89E-03	-0.828
PE(18:1_18:2)	Negative	4.13E-04	-1.792
PE(18:2/18:2)	Negative	3.77E-02	-1.269
PE(P-18:1/16:1)	Negative	1.46E-02	-0.640
PE(P-18:1/22:6)	Negative	4.08E-02	-0.597
PG(16:0_18:1)	Negative	8.85E-03	-0.764
PG(34:3)	Negative	1.65E-02	-1.298
PI(16:0_18:1)	Negative	2.84E-02	-0.709
PI(16:0_18:2)	Negative	1.90E-02	-0.945
PI(16:1_18:1)	Negative	8.78E-04	-1.763
PS(16:1_22:1)	Negative	4.25E-02	-0.894
PS(18:0_18:1)	Negative	2.49E-02	-0.855
LPE(18:1/0:0)	Positive	2.66E-02	-1.087
PC(15:0_18:2)	Positive	7.07E-03	-1.006
PC(16:0_20:5)+	Positive	1.75E-03	-1.364
PC(17:0_18:2)_A	Positive	3.78E-03	-0.967
PC(17:0_18:3)_A	Positive	4.64E-03	-0.906
PC(17:0_20:4)	Positive	4.48E-03	-0.818
PC(19:0_20:4)	Positive	2.09E-02	-0.797
PC(28:0)	Positive	6.51E-03	-0.950
PC(30:1)_A	Positive	7.39E-03	-1.522
PC(30:1)_B	Positive	1.65E-02	-0.900
PC(34:3)_A	Positive	9.93E-04	-2.083
PC(34:3)_B	Positive	9.48E-05	-1.248
PC(34:3)_C	Positive	3.25E-03	-0.925
PC(35:6)	Positive	7.89E-03	-0.815
PE(16:0_18:2)+	Positive	1.53E-04	-1.266
PE(18:0_18:1)	Positive	2.34E-03	-0.889

Table S3. Continued

Lipid	Ionization Mode	p-value	Log_2FC
PE(18:0_18:2)+	Positive	1.23E-02	-0.976
PE(18:1_18:2)_A	Positive	1.53E-05	-2.175
PE(18:1_18:2)_B	Positive	9.56E-08	-1.740
PE(38:5)	Positive	3.99E-03	-1.104
SM(d16:1/24:1)	Positive	2.58E-02	-0.789
SM(d18:1/22:1);SM(d18:2/22:0)	Positive	4.34E-02	-1.010
SM(d18:1/24:1)_A	Positive	1.97E-02	-0.800

Table S4. Statistically significant upregulated lipids for ebs3 versus pluripotent comparison of the neural stem cell lineage.

Lipid	Ionization Mode	p-value	Log_2FC
FA 20:1	Negative	9.47E-04	1.395
FA 20:2	Negative	5.23E-03	1.126
FA 22:4	Negative	3.63E-03	1.186
FA 22:5	Negative	2.43E-04	2.423
FA 22:6	Negative	1.73E-03	0.847
FA 24:1	Negative	2.67E-03	0.901
LPE(O-18:1_0:0)	Negative	6.28E-03	1.572
PC(0:0/18:1)	Negative	1.23E-02	1.012
PC(0:0/20:3)	Negative	8.14E-03	1.356
PC(0:0/20:4)	Negative	1.46E-02	2.008
PE(0:0/20:4)	Negative	2.85E-02	1.248
PE(P-16:0/20:4)	Negative	2.45E-03	0.733
PE(P-16:0/20:5)	Negative	5.02E-06	1.839
PE(P-16:0/22:5); PE(P-18:1/20:4)	Negative	4.63E-03	0.999
PE(P-18:0/20:4)	Negative	1.83E-03	1.094
PE(P-18:1/20:5)	Negative	1.71E-04	2.471
PS(18:0_22:3)	Negative	1.94E-02	0.879
PC(O-16:0/20:5);PC(P-16:0/20:4)	Positive	1.90E-05	1.763
PC(O-16:0/22:5);PC(P-18:0/20:4)_A	Positive	1.32E-02	1.371
PC(O-18:0/22:5);PC(P-18:0/22:4)	Positive	5.65E-03	1.129
PC(O-38:4);PC(P-38:3)_A	Positive	4.19E-02	0.695
PC(O-40:6);PC(P-40:5)_A	Positive	1.38E-04	0.956
PC(P-16:0/16:0)	Positive	4.23E-04	1.354
PC(P-16:0/18:1);PC(P-18:0/16:1)	Positive	3.54E-02	0.794
PC(P-16:0/22:5)	Positive	2.51E-04	1.316
PC(P-18:0/20:4)_B	Positive	4.68E-06	1.170
TG(14:0_18:1_18:1)	Positive	3.09E-02	0.784
TG(16:0_16:1_20:4);TG(16:0_18:2_18:3)	Positive	7.98E-03	1.888
TG(16:0_18:0_18:1)	Positive	3.39E-02	0.898
TG(16:0_18:3_22:4)	Positive	6.97E-03	2.163
TG(16:0_20:2_22:4);TG(18:0_18:0_22:6)	Positive	2.30E-04	1.884
TG(16:1_16:1_18:3)	Positive	1.82E-03	2.059
TG(16:1_18:1_20:4);TG(18:2/18:2/18:2)	Positive	6.57E-03	2.251
TG(18:0_18:0_18:1)	Positive	1.76E-03	1.511
TG(18:0_18:0_20:5);TG(18:0_18:1_20:4)	Positive	3.05E-03	1.653
TG(18:0_18:1_18:1)	Positive	2.27E-03	0.942

Table S4. continued

Lipid	Ionization Mode	p-value	Log_2FC
TG(18:0_18:0_20:5);TG(18:0_18:1_20:4)	Positive	3.05E-03	1.653
TG(18:0_18:1_18:1)	Positive	2.27E-03	0.942
TG(18:0_18:1_20:1)	Positive	1.35E-04	1.631
TG(18:1_18:1_20:4)	Positive	1.49E-02	2.327
TG(18:1_18:2_20:4)	Positive	3.80E-03	2.027
TG(18:1_20:1_20:1)	Positive	9.53E-05	1.739
TG(18:1_20:4_20:4)	Positive	1.30E-03	2.212
TG(54:4)	Positive	2.66E-03	1.799
TG(54:7)_A	Positive	1.40E-02	1.669
TG(56:4)_A	Positive	9.74E-03	1.231
TG(58:2)	Positive	1.20E-03	1.740
TG(58:4)	Positive	1.38E-05	2.174
TG(58:5)_A	Positive	1.66E-04	1.904
TG(58:7)_A	Positive	1.23E-04	2.283
TG(58:8)_A	Positive	2.34E-05	2.588

Table S5. Statistically significant downregulated lipids for ebs7 versus ebs3 comparison of the neural stem cell lineage.

Lipid	Ionization Mode	p-value	Log_2FC
CL(68:4)	Negative	1.65E-02	-0.422
PC(14:0_18:2)	Negative	3.03E-02	-0.436
PE(16:1_18:1)	Negative	3.04E-03	-0.677
PE(P-18:1/16:1)	Negative	1.78E-02	-0.608
PI(16:0_18:1)	Negative	7.75E-03	-1.185
PI(16:1_18:1)	Negative	3.80E-02	-1.009
PC(15:0_18:2)	Positive	1.99E-02	-0.865
PC(17:0_18:2)_A	Positive	1.53E-02	-0.775
PC(17:0_20:4)	Positive	1.15E-02	-0.706
PE(18:0_18:1)	Positive	2.34E-03	-0.891
PE(18:0_18:2)+	Positive	4.03E-02	-0.810
SM(d16:1/24:1)	Positive	6.60E-04	-1.354
SM(d18:1/22:1);SM(d18:2/22:0)	Positive	1.75E-02	-1.176
PE(P-16:0/18:2)	Negative	3.35E-02	-0.519
PI(18:0_18:1)	Negative	1.04E-02	-1.360
PC(17:0_18:3)_B	Positive	4.00E-02	-0.557
PC(17:0_22:6)	Positive	2.11E-02	-0.655
PC(37:6)_A	Positive	4.31E-02	-1.297
PC(P-18:0/16:0)	Positive	2.85E-02	-0.635
SM(d18:0/18:0)	Positive	3.35E-03	-1.391
SM(d18:1/18:0)	Positive	2.36E-02	-1.089
SM(d18:1/20:0)	Positive	6.02E-03	-1.317
SM(d18:1/20:1)	Positive	2.37E-02	-0.805
TG(16:0_18:0_18:1)	Positive	3.64E-02	-0.846

Table S6. Statistically significant upregulated lipids for ebs7 versus ebs3 comparison of the neural stem cell lineage.

Lipid	Ionization Mode	p-value	Log_2FC
FA 16:2	Negative	4.13E-02	1.327
FA 20:4	Negative	8.62E-03	1.124
FA 22:4	Negative	1.14E-02	0.929
FA 22:5	Negative	3.84E-02	0.942
FA 22:6	Negative	6.75E-05	1.449
FA 24:1	Negative	2.67E-03	0.871
PC(16:0_22:6)	Negative	2.46E-03	1.323
PC(37:5)_A	Positive	3.24E-02	0.824
PC(O-16:0/20:5);PC(P-16:0/20:4)	Positive	1.54E-03	1.144
PC(O-16:0/22:5);PC(P-18:0/20:4)_A	Positive	9.77E-03	1.461
PC(O-18:0/22:5);PC(P-18:0/22:4)	Positive	1.09E-02	1.019
PC(O-38:4);PC(P-38:3)_A	Positive	7.02E-03	0.911
PC(O-40:6);PC(P-40:5)_A	Positive	3.26E-06	1.559
PC(P-16:0/16:0)	Positive	7.25E-03	0.884
PC(P-16:0/18:1);PC(P-18:0/16:1)	Positive	1.95E-03	1.190
PC(P-16:0/22:5)	Positive	9.94E-04	1.136
PC(P-18:0/20:4)_B	Positive	3.14E-05	0.992
PE(P-16:0/22:6)	Negative	4.85E-03	1.320
PE(P-18:1/22:6)	Negative	9.17E-06	1.875
PI(16:0_22:5)	Negative	4.44E-03	0.998
PI(18:1_20:4)	Negative	4.81E-02	0.884
TG(18:1_20:4_20:4)	Positive	3.22E-02	1.367
TG(54:7)_A	Positive	7.18E-03	2.257
TG(56:8)_A	Positive	7.93E-03	2.512
TG(58:8)_A	Positive	5.34E-03	1.188

Table S7. Statistically significant downregulated lipids for nsc21 versus ebs7 comparison of the neural stem cell lineage.

Lipid	Ionization Mode	p-value	Log_2FC
Cer(d18:0/24:1)	Negative	5.27E-03	-1.505
Cer(d18:1/20:0)	Negative	1.53E-04	-1.591
Cer(d18:1/22:0)	Negative	3.76E-04	-1.548
Cer(d18:1/23:0)	Negative	1.59E-02	-1.382
Cer(d18:1/24:1)	Negative	7.48E-03	-0.955
Cer(d18:2/22:0)	Negative	1.96E-04	-2.861
Cer(d18:2/23:0)	Negative	2.42E-05	-2.464
DG(16:0_18:1)	Positive	2.86E-03	-1.053
DG(18:0_18:1)	Positive	6.92E-03	-1.206
DG(18:1_18:1)	Positive	1.81E-03	-1.131
FA 20:1	Negative	1.29E-04	-1.798
FA 20:2	Negative	1.15E-03	-1.462
FA 20:4	Negative	1.32E-03	-1.479
FA 22:1	Negative	2.19E-02	-1.309
FA 22:4	Negative	1.96E-05	-2.337
FA 22:5	Negative	2.27E-03	-1.903
FA 24:1	Negative	2.90E-07	-2.169
LPE(O-18:1_0:0)	Negative	3.59E-03	-1.735
PC(0:0/18:1)	Negative	3.43E-04	-1.703
PC(0:0/20:3)	Negative	9.99E-05	-2.411
PC(0:0/20:4)	Negative	2.53E-04	-3.260
PC(16:0_22:5)	Negative	2.40E-02	-1.132
PC(O-16:0/20:5);PC(P-16:0/20:4)	Positive	1.61E-06	-2.177
PC(O-16:0/22:5);PC(P-18:0/20:4)_A	Positive	1.58E-04	-2.283
PC(O-40:6);PC(P-40:5)_A	Positive	7.22E-03	-0.592
PC(P-16:0/16:0)	Positive	4.23E-04	-1.558
PC(P-16:0/18:1);PC(P-18:0/16:1)	Positive	1.33E-03	-1.264
PC(P-16:0/22:5)	Positive	4.81E-05	-1.537
PC(P-18:0/20:4)_B	Positive	4.87E-05	-0.942
PE(0:0/20:4)	Negative	4.29E-02	-1.157
PE(P-16:0/20:4)	Negative	1.43E-03	-0.959
PE(P-16:0/22:5); PE(P-18:1/20:4)	Negative	1.22E-02	-0.864
PE(P-18:0/20:4)	Negative	4.71E-06	-1.956
PI(18:0_18:1)	Negative	1.04E-02	-1.343
SM(d18:1/18:1)_B	Positive	1.30E-02	-1.014
SM(d18:1/20:1)	Positive	1.87E-02	-0.872
TG(54:4)	Positive	2.10E-04	-2.486

Table S7. continued

Lipid	Ionization Mode	p-value	Log_2FC
TG(12:0_16:0_18:1);TG(14:0_14:0_18:1); TG(14:0_16:0_16:1)	Positive	1.75E-04	-1.354
TG(12:0_16:1_18:1);TG(14:0_14:0_18:2); TG(14:0_16:1_16:1)	Positive	2.53E-02	-1.017
TG(14:0_16:0_18:1);TG(16:0_16:0_16:1)	Positive	3.60E-05	-1.372
TG(14:0_16:0_18:2);TG(14:0_16:1_18:1); TG(16:0_16:1_16:1)	Positive	5.12E-05	-1.665
TG(14:0_16:1_18:2)	Positive	5.02E-04	-1.477
TG(14:0_18:1_18:1)	Positive	3.27E-06	-2.087
TG(14:0_18:1_18:2);TG(16:1_16:1_18:1)	Positive	1.00E-04	-1.930
TG(16:0_16:0_18:1)	Positive	7.89E-04	-1.401
TG(16:0_16:1_20:4);TG(16:0_18:2_18:3)	Positive	7.17E-03	-1.938
TG(16:0_17:0_18:2)	Positive	3.37E-02	-0.987
TG(16:0_18:0_18:1)	Positive	4.81E-06	-2.327
TG(16:0_18:0_18:2);TG(16:0_18:1_18:1)	Positive	2.25E-05	-1.907
TG(16:0_18:1_18:2)	Positive	1.67E-05	-2.099
TG(16:0_18:1_18:3);TG(16:0_18:2_18:2)	Positive	1.31E-03	-1.718
TG(16:0_18:3_22:4)	Positive	8.16E-03	-2.089
TG(16:0_20:2_22:4);TG(18:0_18:0_22:6)	Positive	2.30E-04	-2.107
TG(16:1_16:1_18:2)	Positive	2.27E-03	-2.545
TG(16:1_16:1_18:3)	Positive	1.08E-04	-2.737
TG(16:1_18:1_20:4);TG(18:2/18:2/18:2)	Positive	2.49E-03	-2.545
TG(18:0_18:0_18:1)	Positive	8.09E-07	-3.087
TG(18:0_18:0_20:5);TG(18:0_18:1_20:4)	Positive	1.89E-04	-2.342
TG(18:0_18:1_18:1)	Positive	2.58E-08	-2.589
TG(18:0_18:1_18:2);TG(18:1/18:1/18:1)	Positive	1.36E-04	-1.957
TG(18:0_18:1_20:1)	Positive	5.34E-09	-3.686
TG(18:1_18:1_20:4)	Positive	8.18E-03	-2.559
TG(18:1_18:2_18:2)	Positive	8.64E-03	-1.923
TG(18:1_18:2_20:4)	Positive	4.88E-05	-3.259
TG(18:1_20:1_20:1)	Positive	1.44E-08	-3.532
TG(18:1_20:4_20:4)	Positive	1.54E-03	-2.145
TG(54:7)_A	Positive	1.40E-02	-1.942
TG(56:4)_A	Positive	6.09E-05	-2.259
TG(58:2)	Positive	5.47E-07	-3.663
TG(58:4)	Positive	3.89E-07	-2.949
TG(58:5)_A	Positive	8.68E-06	-2.497
TG(58:7)_A	Positive	5.82E-05	-2.468
TG(58:8)_A	Positive	3.79E-05	-2.460

Table S8. Statistically significant upregulated lipids for nsc21 versus ebs7 comparison of the neural stem cell lineage.

Lipid	Ionization Mode	p-value	Log_2FC
CL(68:4)	Negative	3.08E-05	1.069
LPC(14:0/0:0)	Positive	2.73E-03	1.378
LPE(18:1/0:0)	Positive	1.13E-04	2.094
PC(14:0_16:0)+	Positive	3.66E-02	0.729
PC(14:0_16:1)	Negative	1.29E-04	1.068
PC(14:0_18:2)	Negative	1.06E-07	1.965
PC(14:0_18:2)+	Positive	2.94E-02	1.883
PC(15:0_18:2)	Positive	4.19E-03	1.084
PC(16:0_18:0)+	Positive	3.63E-03	0.875
PC(16:0_20:3)+	Positive	2.28E-02	1.319
PC(16:0_20:4);PC(18:1_18:3)	Positive	4.69E-02	1.084
PC(16:0_20:5)+	Positive	1.15E-02	1.038
PC(17:0_18:2)_B	Positive	1.45E-03	2.019
PC(17:0_18:3)_A	Positive	5.29E-07	2.172
PC(17:0_18:3)_B	Positive	1.29E-04	1.497
PC(17:0_20:4)	Positive	1.10E-06	2.237
PC(17:0_22:6)	Positive	2.72E-08	2.604
PC(18:0_18:1)	Positive	8.50E-03	0.509
PC(18:0_22:4)+	Positive	3.61E-04	1.626
PC(18:1_18:2)+	Positive	2.69E-02	1.377
PC(18:1_22:6)_A	Positive	1.57E-02	1.029
PC(19:0_20:4)	Positive	4.78E-07	2.295
PC(28:0)	Positive	3.45E-05	1.660
PC(30:1)	Positive	3.83E-05	2.703
PC(30:1)_B	Positive	5.62E-06	2.114
PC(34:3)_A	Positive	5.09E-04	2.258
PC(34:3)_B	Positive	9.28E-09	2.613
PC(34:3)_C	Positive	2.41E-06	1.907
PC(35:6)	Positive	3.78E-08	2.520
PC(37:4)	Positive	1.15E-03	1.715
PC(37:6)_A	Positive	1.29E-03	2.015
PC(P-18:0/16:0)	Positive	1.86E-04	1.137
PC(P-32:0);PC(O-32:1)	Positive	4.11E-04	1.202
PE(14:0_16:0)	Negative	2.46E-07	1.832
PE(14:0_18:1);PE(16:0_16:1)	Negative	9.82E-06	3.133
PE(16:0_18:0)	Negative	2.15E-04	1.203
PE(16:0_18:2)+	Positive	1.43E-08	2.715

Table S8. continued

Lipid	Ionization Mode	p-value	Log_2FC
PE(16:0_20:4)	Negative	5.87E-05	1.836
PE(16:0_22:4)	Negative	1.12E-04	1.220
PE(16:0_22:5);PE(18:1_20:4)	Negative	3.43E-02	1.622
PE(16:1/16:1)	Negative	1.74E-06	2.987
PE(16:1_18:1)	Negative	6.49E-08	2.135
PE(18:0_18:1)	Positive	2.58E-04	1.132
PE(18:0_18:2)+	Positive	4.05E-02	0.787
PE(18:0_18:3)	Negative	1.77E-07	1.908
PE(18:0_20:5)	Negative	1.66E-04	2.148
PE(18:0_22:6)	Negative	5.25E-06	2.778
PE(18:1_18:2)	Negative	8.24E-05	2.136
PE(18:1_18:2)_A	Positive	1.53E-05	2.316
PE(18:1_18:2)_B	Positive	5.38E-10	2.564
PE(18:1_22:6)	Negative	7.58E-04	3.857
PE(18:2/18:2)	Negative	6.76E-05	2.961
PE(38:5)	Positive	4.41E-03	0.973
PE(P-16:0/18:2)	Negative	5.64E-06	1.764
PE(P-16:0/18:3)	Negative	4.25E-03	1.897
PE(P-16:0/22:6)	Negative	6.99E-05	2.069
PE(P-18:1/16:1)	Negative	6.82E-05	1.143
PE(P-18:1/22:6)	Negative	4.14E-05	1.625
PI(16:0_18:1)	Negative	1.93E-02	0.765
PI(16:0_18:2)	Negative	9.90E-03	1.442
PI(16:0_20:3)	Negative	1.66E-03	0.971
PI(16:1_18:1)	Negative	1.15E-02	1.240
PI(18:0_18:2)	Negative	3.29E-02	1.650
PI(18:0_18:3)	Negative	2.23E-04	1.084
PI(18:0_22:6)	Negative	2.27E-06	1.971
PS(16:1_22:1)	Negative	4.25E-02	0.844
PS(18:0_18:1)	Negative	1.99E-05	1.861
PS(18:0_22:3)	Negative	6.92E-05	1.992
SM(d16:1/16:0);SM(d18:1/14:0)	Positive	3.41E-02	1.184
SM(d32:0)	Positive	3.07E-02	1.058

Table S9. Statistically significant downregulated lipids for neu42 versus nsc21 comparison of the neural stem cell lineage.

Lipid	Ionization Mode	p-value	Log_2FC
CL(68:3)	Negative	2.75E-03	-2.178
CL(68:4)	Negative	5.60E-08	-1.674
CL(70:4)	Negative	8.78E-06	-1.629
CL(72:4)	Negative	2.05E-03	-1.395
CL(74:6)	Negative	2.37E-02	-1.263
FA 16:1	Negative	3.11E-02	-0.785
FA 20:1	Negative	1.62E-06	-2.463
FA 20:2	Negative	1.15E-03	-1.619
FA 22:6	Negative	7.29E-04	-0.892
FA 24:1	Negative	3.72E-04	-1.085
PC(14:0_16:1)	Negative	1.29E-04	-1.003
PC(14:0_18:2)	Negative	2.63E-04	-0.993
PC(17:0_18:2)_A	Positive	3.28E-06	-1.858
PC(17:0_20:4)	Positive	4.05E-03	-0.787
PC(18:0_20:3)	Negative	3.11E-04	-1.212
PC(19:0_20:4)	Positive	9.00E-06	-1.716
PC(35:6)	Positive	1.16E-02	-0.666
PC(38:2)_A	Positive	3.87E-03	-1.702
PE(14:0_16:0)	Negative	1.06E-06	-1.549
PE(14:0_18:1);PE(16:0_16:1)	Negative	7.93E-03	-1.475
PE(16:0_20:4)	Negative	1.86E-03	-1.215
PE(16:0_22:4)	Negative	1.11E-03	-0.918
PE(16:1/16:1)	Negative	1.63E-03	-1.365
PE(16:1_18:1)	Negative	7.71E-04	-0.762
PE(18:0_18:3)	Negative	1.04E-03	-0.853
PE(P-18:0/20:1)	Negative	1.09E-02	-1.189
PE(P-18:1/20:1)	Negative	3.52E-02	-0.867
PE(P-18:1/22:3)	Negative	9.49E-03	-1.072
PI(16:0_22:5)	Negative	1.41E-02	-0.592
PI(18:0_22:4)	Negative	7.60E-04	-1.124
PI(18:1_20:3)	Negative	3.38E-06	-1.658
PS(16:0_18:1)	Negative	1.71E-04	-3.099
PS(18:0_18:1)	Negative	2.62E-03	-1.120
SM(d18:1/20:1)	Positive	4.08E-02	-0.681
SM(d34:2)	Positive	4.92E-03	-1.528
TG(16:0_20:2_22:4);TG(18:0_18:0_22:6)	Positive	2.30E-04	-2.000

Table S9. continued

Lipid	Ionization Mode	p-value	Log_2FC
TG(18:0_18:0_20:5);TG(18:0_18:1_20:4)	Positive	5.12E-03	-1.423
TG(18:0_18:1_18:1)	Positive	6.15E-04	-1.060
TG(18:0_18:1_20:1)	Positive	1.23E-03	-1.156
TG(18:1_20:1_20:1)	Positive	1.31E-05	-1.978
TG(54:4)	Positive	9.45E-03	-1.407
TG(56:4)_A	Positive	2.60E-04	-1.856
TG(58:4)	Positive	1.38E-05	-2.098
TG(58:5)_A	Positive	2.76E-05	-2.141
TG(58:7)_A	Positive	5.14E-03	-1.419
TG(58:8)_A	Positive	8.77E-03	-1.045

Table S10. Statistically significant upregulated lipids for neu42 versus nsc21 comparison of the neural stem cell lineage.

Lipid	Ionization Mode	p-value	Log_2FC
Cer(d18:0/24:1)	Negative	2.39E-05	2.385
Cer(d18:1/18:0)	Negative	4.79E-05	2.362
Cer(d18:1/20:0)	Negative	9.43E-03	0.958
Cer(d18:1/22:0)	Negative	3.76E-04	1.472
Cer(d18:1/23:0)	Negative	2.90E-02	1.196
Cer(d18:1/24:0)	Negative	1.25E-04	2.416
Cer(d18:1/24:0)+	Positive	3.40E-09	2.717
Cer(d18:1/24:1)	Negative	1.67E-03	1.077
FA 16:2	Negative	4.03E-03	1.666
FA 20:4	Negative	9.49E-04	1.464
HexCer(d18:1/22:0)	Negative	4.15E-02	0.829
HexCer(d18:1/24:0)	Negative	8.31E-06	1.984
LPC(0:0/16:0)	Positive	4.41E-03	0.804
LPC(0:0/18:0)	Positive	1.44E-02	1.959
LPC(0:0/18:2)	Positive	3.93E-04	2.389
LPC(14:0/0:0)	Positive	3.94E-02	0.882
LPC(16:1/0:0)	Positive	2.10E-02	1.520
LPC(18:0/0:0)	Positive	4.06E-05	3.276
LPC(18:1/0:0)	Positive	2.96E-02	1.228
LPC(22:5_0:0)	Positive	2.13E-04	2.566
LPE(0:0/18:1)	Positive	2.24E-04	1.990
PC(0:0/18:0)	Negative	5.94E-05	2.689
PC(0:0/18:1)	Negative	1.23E-02	1.063
PC(15:0_20:4);PC(17:2_18:2)	Positive	1.53E-02	1.232
PC(16:0/0:0)	Negative	2.80E-02	0.929
PC(16:0_18:0)+	Positive	5.92E-04	1.018
PC(16:0_20:5)+	Positive	3.14E-02	0.814
PC(16:0_22:4)+	Positive	1.19E-03	1.476
PC(17:0_18:3)_B	Positive	2.94E-04	1.299
PC(17:0_22:6)	Positive	2.11E-02	0.659
PC(18:0_18:1)	Positive	2.12E-05	0.906
PC(18:1_20:3)_A	Positive	6.36E-04	1.636
PC(34:3)_C	Positive	1.55E-05	1.540
PC(37:5)_A	Positive	3.01E-04	1.551
PC(O-16:0/20:5);PC(P-16:0/20:4)	Positive	2.89E-04	1.304
PC(O-36:3);PC(P-36:2)_A	Positive	7.91E-03	1.109

Table S10. continued

Lipid	Ionization Mode	p-value	Log_2FC
PC(O-36:4);PC(P-36:3)	Positive	2.33E-03	0.915
PC(O-40:6);PC(P-40:5)_A	Positive	1.34E-02	0.507
PC(P-16:0/16:0)	Positive	4.23E-04	1.487
PC(P-16:0/18:1);PC(P-18:0/16:1)	Positive	2.18E-05	1.784
PC(P-16:0/22:5)	Positive	5.57E-03	0.875
PC(P-18:0/20:4)_B	Positive	1.05E-02	0.482
PE(0:0/18:0)	Negative	2.21E-02	0.960
PE(0:0/18:1)	Negative	2.57E-02	1.337
PE(18:0_18:1)	Positive	3.72E-05	1.290
PE(18:0_22:6)	Negative	2.62E-03	1.440
PE(P-16:0/18:3)	Negative	4.25E-03	1.781
PE(P-18:0/18:2)	Negative	3.61E-03	0.944
PE(P-18:1/18:1)	Negative	4.02E-04	1.149
PE(P-18:1/22:6)	Negative	4.08E-02	0.628
PI(16:0_18:1)	Negative	7.75E-03	1.110
PI(18:0_18:1)	Negative	2.49E-05	2.399
SM(d16:1/22:3);SM(d18:1/20:3)	Positive	9.14E-03	2.659
SM(d18:0/18:0)	Positive	3.38E-03	1.302
SM(d18:1/18:0)	Positive	3.73E-05	2.147
SM(d18:1/18:1)_B	Positive	1.26E-03	1.268
SM(d18:1/20:0)	Positive	3.60E-02	0.941
SM(d18:1/22:0)	Positive	2.19E-05	2.292
SM(d18:1/24:0)	Positive	1.70E-06	4.099
SM(d18:1/24:1)_A	Positive	6.61E-03	1.114
SM(d18:2/18:0)	Positive	6.10E-03	1.515
TG(14:0_16:1_18:2)	Positive	1.05E-02	1.008
TG(16:0_17:0_18:1)	Positive	4.85E-02	1.020
TG(16:1_16:1_18:2)	Positive	7.15E-03	2.107
TG(16:1_16:1_18:3)	Positive	3.11E-05	2.946

Table S11. Statistically significant downregulated lipids for ebs3 versus pluri comparison of the mesodermal stem cell lineage.

Lipid	Ionization Mode	p-value	Log_2FC
HexCer(d18:1/22:0)	Negative	3.56E-02	-0.785
HexCer(d18:1/24:0)	Negative	2.69E-03	-1.353
LPE(0:0/22:6)	Positive	1.83E-03	-1.709
PC(14:0_16:0)+	Positive	1.80E-02	-0.557
PC(14:0_18:1);PC(16:0_16:1)_A	Positive	3.05E-02	-0.579
PC(14:0_18:2)	Negative	1.22E-02	-1.388
PC(14:0_18:2)_A	Positive	1.08E-04	-2.441
PC(14:0_18:2)_B	Positive	3.15E-05	-1.839
PC(16:0_18:2)_A+	Positive	1.04E-03	-0.868
PC(16:0_18:2)_C	Negative	2.77E-02	-0.558
PC(16:0_18:3)_A+	Positive	2.51E-02	-1.417
PC(16:0_20:3)	Negative	3.67E-02	-0.976
PC(16:1_18:2)	Negative	1.09E-02	-1.336
PC(16:1_18:2)+	Positive	3.66E-03	-1.494
PC(18:0_20:4)+	Positive	3.53E-02	-0.927
PC(18:0_22:4)_B+	Positive	6.59E-03	-1.452
PC(18:1_18:2)	Positive	1.30E-03	-0.883
PC(18:1_18:2)_A	Negative	2.40E-03	-0.995
PC(28:0)	Positive	5.46E-04	-1.663
PC(30:1)_A	Positive	7.90E-06	-2.073
PC(30:1)_B	Positive	5.05E-04	-1.476
PE(16:0_18:2)_A	Negative	8.20E-05	-1.476
PE(16:0_18:2)_A+	Positive	7.22E-05	-1.762
PE(16:0_18:2)_B	Negative	1.12E-05	-1.221
PE(16:0_18:2)_B+	Positive	5.92E-04	-1.497
PE(16:0_18:3)_A	Negative	1.34E-06	-3.417
PE(18:0_20:5)	Negative	2.24E-04	-2.585
PE(18:0_22:6)	Negative	1.33E-03	-2.018
PE(18:1_18:2)_A+	Positive	1.07E-05	-1.970
PE(18:1_18:2)_B	Negative	1.88E-03	-1.457
PE(18:1_18:2)_B+	Positive	3.66E-05	-2.149
PE(18:1_20:4)	Positive	3.40E-02	-0.795
PE(18:1_20:5)	Positive	1.72E-05	-3.024
PE(19:0_20:2)	Negative	3.63E-02	-1.754
PE(32:1)_A	Negative	1.06E-02	-2.112
PE(32:1)_C	Negative	1.18E-06	-1.426
PE(36:5)	Negative	1.59E-03	-2.534

Table S11. continued

Lipid	Ionization Mode	p-value	Log_2FC
PE(P-16:0/18:1)	Negative	4.24E-03	-1.818
PE(P-16:0/18:2)	Negative	1.31E-04	-1.823
PE(P-16:0/20:5)	Negative	9.51E-04	-1.445
PE(P-18:0/18:2)	Negative	1.45E-02	-1.353
PG(34:1)	Negative	1.29E-02	-1.843
PI(16:0_18:1)	Negative	1.19E-03	-2.459
PI(16:0_18:2)	Negative	2.09E-03	-2.964
PI(16:0_20:3)	Negative	2.28E-05	-1.268
PI(18:0_18:2)	Negative	2.98E-02	-2.965
PI(18:0_18:3)	Negative	1.71E-05	-1.585
PI(18:1_18:2)	Negative	1.28E-02	-1.612
PI(18:1_20:3)	Negative	3.49E-03	-3.687
PS(16:1_22:1)	Negative	2.79E-05	-1.163
PS(18:0_18:2)	Negative	6.93E-03	-1.200
SM(d16:1/16:0);SM(d18:1/14:0)	Positive	1.09E-02	-0.605
SM(d16:1/18:3)_B	Positive	3.77E-02	-1.070
SM(d18:2/16:0)+	Positive	1.71E-02	-0.951
SM(d42:3)+	Positive	1.32E-02	0.286

Table S12. Statistically significant upregulated lipids for ebs3 versus pluri comparison of the mesodermal stem cell lineage.

Lipid	Ionization Mode	p-value	Log_2FC
FA 13:0	Negative	4.60E-02	1.934
FA 18:2	Negative	3.52E-02	0.919
FA 20:0_A	Negative	2.21E-02	1.927
FA 20:4	Negative	3.46E-02	1.678
FA 22:0	Negative	3.49E-02	1.093
FA 24:0_B	Negative	1.09E-02	1.215
PC(16:0_18:1)	Negative	4.46E-02	0.579
PC(18:0_22:5)_B	Negative	2.41E-02	1.152
PC(O-16:0/20:5);PC(P-16:0/20:4)	Positive	1.49E-03	1.941
PC(O-16:0/22:5);PC(P-18:0/20:4)_A	Negative	2.44E-02	1.397
PC(O-16:0/22:6)	Negative	4.77E-04	2.774
PC(O-18:0/20:4)	Negative	1.48E-02	1.638
PC(P-16:0/20:4)	Negative	9.71E-05	3.048
PC(P-16:0/22:5)	Positive	4.21E-03	1.958
PC(P-18:0/20:4)_B	Negative	7.35E-03	2.704
PC(P-18:0/20:4)_B+	Positive	4.74E-02	1.160
PC(P-18:0/20:5)	Positive	4.54E-03	1.857
PE(P-16:0/20:4)	Negative	3.81E-02	0.410
PE(P-18:0/20:4)	Negative	6.80E-03	0.791
PE(P-18:0/22:4)	Negative	8.94E-03	0.715
SM(d18:0/18:1)	Negative	1.12E-03	1.107
SM(d18:1/18:0)	Positive	4.25E-02	0.442
TG(16:0_16:1_20:4); TG(16:0_18:2_18:3)	Positive	9.06E-03	1.391
TG(16:0_18:1_20:4)	Positive	2.99E-04	1.406
TG(16:0_18:2_19:0)	Positive	2.77E-02	0.576
TG(16:0_20:2_22:4); TG(18:0_18:0_22:6)	Positive	6.31E-05	1.411
TG(18:1_18:1_20:4)	Positive	1.62E-04	1.940
TG(18:1_18:2_18:2)	Positive	1.28E-04	1.456
TG(18:1_18:2_20:4)	Positive	8.53E-03	2.181
TG(53:3)	Positive	1.86E-02	0.722
TG(53:4)	Positive	1.58E-02	0.805
TG(54:6)	Positive	2.26E-03	2.235
TG(56:4)_B	Positive	3.70E-02	0.672
TG(58:4)_B	Positive	2.27E-02	0.995
TG(58:5)_A	Positive	2.28E-04	1.022

Table S12. continued

Lipid	Ionization Mode	p-value	Log_2FC
TG(58:7)_A	Positive	2.24E-04	1.936
TG(58:8)_A	Positive	3.84E-02	2.384
TG(58:8)_B	Positive	1.27E-03	1.938
TG(60:10)_A	Positive	2.34E-02	2.340
TG(60:10)_B	Positive	3.41E-03	2.083

Table S13. Statistically significant downregulated lipids for ebm7 versus ebs3 comparison of the mesodermal stem cell lineage.

Lipid	Ionization Mode	p-value	Log_2FC
PC(14:0_18:1);PC(16:0_16:1)_C	Positive	1.90E-02	-1.345
PC(16:0_18:2)_C	Negative	4.34E-05	-1.094
PC(16:0_20:3)	Negative	4.85E-02	-0.914
PC(18:0_22:4)_A	Negative	4.92E-03	-1.314
PC(18:0_22:4)_A+	Positive	1.67E-02	-0.983
PC(18:0_22:5)_B	Negative	6.72E-03	-1.358
PC(30:1)_A	Positive	7.09E-03	-0.843
PC(30:1)_B	Positive	2.78E-04	-1.570
PE(32:1)_C	Negative	3.12E-02	-0.467
PG(16:1_18:1)_A	Negative	1.13E-02	-1.161

Table S14. Statistically significant upregulated lipids for ebm7 versus ebs3 comparison of the mesodermal stem cell lineage.

Lipid	Ionization Mode	p-value	Log_2FC
DG(18:1_18:2)	Positive	2.03E-02	1.798
HexCer(d18:1/24:0)	Negative	2.14E-02	1.027
LPE(0:0/22:6)	Positive	1.08E-02	1.373
PC(18:1_18:2)	Positive	4.21E-06	1.417
PC(18:1_18:2)_A	Negative	6.48E-05	1.816
PC(O-16:0/18:3);PC(P-16:0/18:2)	Negative	2.24E-02	1.677
PE(16:0_18:3)_A	Negative	9.69E-04	1.927
PE(18:0_18:2)_B	Positive	3.03E-02	0.999
PE(18:0_22:5)_B	Negative	4.27E-02	1.154
PE(18:0_22:6)	Negative	5.19E-03	1.722
PE(18:1_18:2)_A+	Positive	1.88E-04	1.560
PE(P-18:0/20:4)	Negative	3.64E-02	0.441
PE(P-18:0/22:6)	Negative	8.58E-03	1.916
PI(18:0_18:2)	Negative	1.02E-02	1.467
PI(18:1_18:2)	Negative	7.42E-03	1.698
PS(16:1_22:1)	Negative	3.14E-02	1.816
TG (16:0_18:0_19:1)	Positive	4.69E-02	1.033
TG(12:0_12:0_18:1);TG(12:0_14:0_16:1); TG(12:0_14:1_16:0)	Positive	1.32E-02	1.662
TG(12:0_14:0_18:1);TG(12:0_16:0_16:1)	Positive	1.52E-02	0.846
TG(12:0_14:0_18:2);TG(12:0_16:1_16:1)	Positive	2.74E-02	2.083
TG(12:0_16:1_18:1);TG(14:0_14:0_18:2); TG(14:0_16:1_16:1)	Positive	2.07E-02	0.540
TG(14:0_16:1_18:2)	Positive	2.88E-03	1.282
TG(14:0_18:1_18:2);TG(16:1_16:1_18:1)	Positive	3.40E-02	0.912
TG(16:0_16:0_18:0)	Positive	1.14E-02	1.124
TG(16:0_16:0_18:1)	Positive	8.03E-03	1.162
TG(16:0_16:1_20:4);TG(16:0_18:2_18:3)	Positive	9.06E-03	1.081
TG(16:0_17:0_18:1)	Positive	3.04E-04	1.435
TG(16:0_17:0_18:2)	Positive	1.25E-02	1.232
TG(16:0_17:1_18:1)	Positive	1.04E-02	1.191
TG(16:0_18:0_18:1)	Positive	2.13E-04	1.057
TG(16:0_18:0_18:2);TG(16:0_18:1_18:1)	Positive	5.48E-03	0.784
TG(16:0_18:1_18:2)	Positive	8.94E-03	0.815
TG(16:0_18:1_18:3);TG(16:0_18:2_18:2)	Positive	2.25E-03	1.074
TG(16:0_18:1_20:4)	Positive	2.27E-02	0.878
TG(16:0_18:2_19:0)	Positive	1.02E-06	1.617

Table S14. continued

Lipid	Ionization Mode	p-value	Log_2FC
TG(16:1_16:1_18:2)	Positive	1.62E-02	1.145
TG(16:1_18:1_20:4);TG(18:2/18:2/18:2)	Positive	8.36E-03	1.659
TG(16:1_18:2_18:3)	Positive	7.46E-03	1.537
TG(18:0_18:1_18:1)	Positive	3.94E-03	0.700
TG(18:0_18:1_18:2);TG(18:1/18:1/18:1)	Positive	2.20E-02	0.642
TG(18:1_18:2_18:2)	Positive	1.28E-04	1.465
TG(18:1_18:2_20:4)	Positive	1.10E-02	2.094
TG(53:3)	Positive	1.29E-04	1.229
TG(53:4)	Positive	3.48E-04	1.328
TG(54:4)	Positive	2.52E-04	1.348
TG(54:7)_A	Positive	4.92E-02	1.856

Table S15. Statistically significant downregulated lipids for ebm14 versus ebm7 comparison of the mesodermal stem cell lineage.

Lipid	Ionization Mode	p-value	Log_2FC
CL(70:4)	Negative	2.93E-02	-2.113
CL(72:4)	Negative	2.48E-02	-2.200
CL(72:5)	Negative	3.14E-02	-2.234
PC(18:0_22:4)_A	Negative	3.87E-02	-0.915
PC(18:0_22:4)_A+	Positive	4.19E-02	-0.782
PC(38:2)	Positive	2.86E-02	-0.697
PC(O-18:0/16:0)	Positive	2.86E-02	-0.820
PC(O-38:4);PC(P-38:3)_A	Positive	1.36E-03	-0.791
PC(P-16:0/16:0)_B	Positive	3.04E-02	-1.060
PC(P-18:0/16:0)+	Positive	1.57E-02	-0.744
PC(P-32:0);PC(O-32:1)_B	Positive	1.82E-02	-0.945
PE(32:1)_C	Negative	2.47E-02	-0.489
PE(P-18:0/20:3)_A	Negative	2.30E-02	-0.649
PG(16:1_18:1)_A	Negative	1.13E-02	-1.363
PG(18:0_18:2)	Negative	2.21E-02	-1.374
PG(18:0_18:2);PG(18:1/18:1)	Negative	1.68E-02	-1.676
TG(16:0_18:0_18:1)	Positive	4.74E-02	-0.384
TG(56:4)_B	Positive	3.60E-02	-0.686
TG(58:5)_A	Positive	3.97E-02	-0.572

Table S16. Statistically significant upregulated lipids for ebm14 versus ebm7 comparison of the mesodermal stem cell lineage.

Lipid	Ionization Mode	p-value	Log_2FC
LPC(0:0/18:0)	Negative	3.82E-02	2.722
LPC(16:0/0:0)	Negative	2.37E-03	2.497
LPC(16:0/0:0)+	Positive	1.87E-02	1.839
PC(15:0_16:0)	Negative	7.67E-03	0.941
PC(16:1_18:2)	Negative	1.19E-02	1.309
PC(16:1_18:2)+	Positive	3.08E-02	1.133
PC(18:1_18:2)_A	Negative	1.25E-02	0.782
PC(30:1)_A	Positive	7.65E-03	0.834
PE(16:0_18:3)_A	Negative	4.16E-03	1.157
PE(18:0_20:3)_A	Negative	4.47E-02	0.447
PE(18:0_20:5)	Negative	2.65E-03	1.996
PE(18:0_22:5)_B	Negative	1.02E-02	1.519
PE(18:0_22:6)	Negative	6.01E-05	2.702
PS(16:1_22:1)	Negative	3.14E-02	1.418
PS(18:0_18:2)	Negative	4.34E-02	0.633
SM(d16:0/16:1);SM(d18:1/14:0)	Negative	9.60E-03	1.080
SM(d16:1/18:3)_B	Positive	3.77E-02	0.647
SM(d18:1/22:0)	Negative	3.75E-02	1.299
SM(d18:1/24:0)	Negative	5.01E-05	3.521
SM(d18:1/24:1)_A	Negative	7.57E-03	0.899
SM(d42:3)	Negative	6.25E-03	1.163

Table S17. Statistically significant downregulated lipids for ebm21 versus ebm14 comparison of the mesodermal stem cell lineage.

Lipid	Ionization Mode	p-value	Log_2FC
Cer(d18:1/18:0)	Negative	1.98E-03	-1.972
Cer(d18:1/20:0)	Negative	6.63E-03	-1.624
FA 12:0	Negative	5.90E-03	-1.257
FA 18:2	Negative	3.52E-02	-1.067
FA 22:0	Negative	3.49E-02	-1.021
FA 24:0_B	Negative	1.09E-02	-1.083
LPC(0:0/20:4)+	Positive	4.40E-02	-1.801
PC(16:0/16:0)	Negative	5.33E-03	-1.307
PC(16:0_18:0)	Negative	1.29E-02	-0.955
PC(16:0_18:1)	Negative	4.46E-02	-0.890
PC(16:0_20:4)	Negative	4.21E-02	-1.554
PC(16:0_22:4)_B	Negative	2.13E-03	-1.339
PC(16:0_22:5);PC(18:1_20:4)	Negative	1.47E-03	-1.748
PC(16:0_22:6)	Positive	5.81E-03	-1.697
PC(18:0_20:3)_B	Negative	5.42E-04	-1.669
PC(18:0_20:4)	Negative	2.79E-03	-2.261
PC(18:0_22:4)_A	Negative	2.38E-02	-1.013
PC(18:0_22:4)_B	Negative	2.12E-02	-1.558
PC(18:0_22:5)_B	Negative	5.97E-04	-1.764
PC(18:1_18:2)_A	Negative	2.35E-03	-1.302
PC(18:1_20:4)	Positive	9.07E-03	-1.167
PC(O-16:0/18:3);PC(P-16:0/18:2)	Negative	8.85E-03	-1.579
PC(O-16:0/20:4);PC(P-18:0/18:3)	Negative	1.52E-03	-1.691
PC(O-16:0/20:5);PC(P-16:0/20:4)	Positive	3.02E-04	-2.257
PC(O-16:0/22:5);PC(P-18:0/20:4)_A	Negative	7.58E-04	-2.030
PC(O-16:0/22:5);PC(P-18:0/20:4)_A+	Positive	2.80E-03	-1.212
PC(O-16:0/22:6)	Negative	1.36E-05	-3.735
PC(O-18:0/20:4)	Negative	1.38E-03	-2.144
PC(O-18:0/22:4)	Negative	4.69E-03	-2.788
PC(O-18:0/22:5)	Negative	1.12E-03	-2.810
PC(O-18:0/22:5);PC(P-18:0/22:4)	Positive	4.33E-02	-1.092
PC(O-38:4);PC(P-38:3)_A	Positive	4.97E-06	-1.338
PC(O-38:4);PC(P-38:3)_B	Positive	7.29E-03	-0.968
PC(P-16:0/18:1)	Negative	4.53E-02	-1.508
PC(P-16:0/20:4)	Negative	2.13E-05	-3.451
PC(P-16:0/22:5)	Positive	2.23E-03	-2.120
PC(P-18:0/20:4)_B	Negative	1.49E-04	-3.949

Table S17. continued

Lipid	Ionization Mode	p-value	Log_2FC
PC(P-18:0/20:4)_B+	Positive	8.89E-05	-2.235
PC(P-18:0/20:5)	Positive	1.87E-03	-2.063
PE(18:0_20:4)	Negative	1.02E-02	-1.070
PE(18:0_22:5)_A	Negative	3.50E-02	-1.242
PE(O-40:5);PE(P-40:4)	Negative	4.18E-05	-1.541
PE(P-16:0/20:4)	Negative	3.23E-03	-0.830
PE(P-16:0/22:4)	Negative	8.57E-05	-1.148
PE(P-16:0/22:5)_A	Negative	1.90E-03	-0.888
PE(P-18:0/20:3)_A	Negative	1.16E-03	-0.930
PE(P-18:0/20:4)	Negative	2.76E-04	-1.112
PE(P-18:0/22:4)	Negative	7.03E-04	-1.151
PS(18:0_22:3)	Negative	1.91E-03	-1.865
SM(d16:1/18:3)_A	Positive	3.47E-02	-1.262
SM(d18:0/18:1)	Negative	1.78E-04	-1.374
SM(d18:1/18:0)	Positive	4.25E-02	-0.571
SM(d18:1/18:1)	Negative	1.38E-05	-1.828
SM(d18:1/20:1)	Positive	3.99E-03	-1.252
SM(d18:2/18:0)	Positive	8.77E-05	-1.329
TG(16:0_16:1_20:4);<br>TG(16:0_18:2_18:3)	Positive	9.06E-03	-1.396
TG(16:0_18:1_20:4)	Positive	1.57E-03	-1.198
TG(16:0_20:2_22:4);<br>TG(18:0_18:0_22:6)	Positive	3.68E-05	-1.483
TG(16:1_18:1_20:4);<br>TG(18:2/18:2/18:2)	Positive	4.97E-02	-1.306
TG(16:1_18:2_18:3)	Positive	4.70E-03	-1.632
TG(18:1_18:1_20:4)	Positive	1.10E-02	-1.258
TG(18:1_18:2_18:2)	Positive	3.91E-03	-0.816
TG(18:1_18:2_20:4)	Positive	1.19E-02	-2.045
TG(18:1_20:4_20:4)	Positive	4.09E-02	-2.581
TG(54:6)	Positive	3.66E-03	-2.112
TG(56:4)_A	Positive	1.17E-02	-0.806
TG(56:4)_B	Positive	4.58E-03	-0.889
TG(56:8)_A	Positive	2.28E-03	-3.021
TG(58:4)_B	Positive	1.63E-04	-1.676
TG(58:5)_A	Positive	7.65E-07	-1.600
TG(58:7)_A	Positive	1.97E-03	-1.566
TG(58:8)_A	Positive	3.15E-02	-2.471
TG(58:8)_B	Positive	7.69E-04	-2.091

Table S17. continued

Lipid	Ionization Mode	p-value	Log_2FC
TG(60:10)_A	Positive	1.15E-02	-2.573
TG(60:10)_B	Positive	2.78E-02	-1.607

Table S18. Statistically significant upregulated lipids for ebm21 versus ebm14 comparison of the mesodermal stem cell lineage.

Lipid	Ionization Mode	p-value	Log_2FC
Cer(d18:1/24:0)+	Positive	4.17E-02	2.173
HexCer(d18:1/22:0)	Negative	2.52E-02	0.879
HexCer(d18:1/24:0)	Negative	1.31E-06	2.579
HexCer(d18:1/24:1)	Negative	1.73E-02	0.876
LPE(0:0/18:1)+	Positive	1.95E-02	2.232
LPE(0:0/22:6)	Positive	2.60E-03	1.631
PC(14:0_18:2)_A	Positive	3.37E-02	1.299
PC(16:0_18:3)_A+	Positive	4.13E-02	1.315
PC(17:0_18:2)_B	Positive	3.06E-02	0.873
PC(28:0)	Positive	1.64E-02	1.158
PC(30:1)_A	Positive	3.33E-04	1.226
PC(30:1)_B	Positive	5.05E-04	1.208
PE(16:0_18:2)_A	Negative	4.03E-02	0.767
PE(16:0_18:2)_A+	Positive	1.54E-02	1.046
PE(16:0_18:2)_B+	Positive	3.73E-02	0.917
PE(16:0_18:3)_A	Negative	2.06E-03	1.637
PE(18:0_18:2)_B	Positive	3.98E-03	1.296
PE(18:1_18:2)_A+	Positive	3.10E-02	0.884
PE(18:1_18:2)_B+	Positive	2.27E-03	1.129
PE(32:1)_A	Negative	4.28E-02	1.158
PE(32:1)_C	Negative	2.38E-02	0.493
PE(P-16:0/18:2)	Negative	1.72E-03	1.393
PE(P-18:0/18:2)	Negative	2.54E-02	1.314
PI(16:0_18:1)	Negative	7.48E-04	1.955
PI(18:0_18:1)_A	Negative	6.49E-03	1.324
PI(18:0_18:1)_B	Negative	7.64E-03	1.379
PI(18:0_18:2)_A	Negative	3.03E-02	1.024
SM(d16:1/18:3)_B	Positive	3.77E-02	0.603
SM(d18:1/22:0)+	Positive	9.02E-03	1.316
SM(d18:1/24:0)	Negative	1.78E-02	1.927
TG(12:0_16:0_18:1);TG(14:0_14:0_18:1); TG(14:0_16:0_16:1)	Positive	2.57E-03	1.386
TG(12:0_14:0_18:1);TG(12:0_16:0_16:1)	Positive	1.57E-02	0.842
TG(12:0_16:1_18:1);TG(14:0_14:0_18:2); TG(14:0_16:1_16:1)	Positive	2.15E-02	0.537
TG(16:0_17:1_18:1)	Positive	1.04E-02	0.886
